# From Mild Discomfort to Myopericarditis—A Complication of Patent Foramen Ovale Occluder Device Placement: A Case Report

**DOI:** 10.1016/j.jscai.2025.104044

**Published:** 2025-11-13

**Authors:** Anastasia Proshkina, Jonathan Shpigelman, Rami Al Ayyubi, Mohamad Bahrou, Ra’ad Al Tamimi, Ahmad Al Turk

**Affiliations:** a Department of Internal Medicine, Southern Illinois University School of Medicine, Springfield, Illinois; b Division of Cardiology, Southern Illinois University School of Medicine, Springfield, Illinois

**Keywords:** case report, myopericarditis, nickel hypersensitivity, patent foramen ovale closure, procedural complications, septal occluder

## Abstract

A 43-year-old woman underwent successful elective closure of a large aneurysmal patent foramen ovale (PFO) using a GORE CARDIOFORM Septal Occluder. She experienced mild chest discomfort immediately postprocedure but was discharged after a transthoracic echocardiogram confirmed appropriate device positioning and no acute abnormalities. She returned the following day with worsening pleuritic chest pain unresponsive to acetaminophen and ibuprofen. Her clinical presentation, together with her electrocardiogram, laboratory, and imaging findings, was diagnostic of myopericarditis. She was treated with colchicine and ibuprofen, resulting in complete symptom resolution. This case illustrates a rare complication of PFO closure, potentially related to nickel hypersensitivity. Currently, no validated tools exist to predict such hypersensitivity reactions, and nickel-free PFO closure devices are not commercially available. Until alternatives become available, clinicians must balance the risk of hypersensitivity reactions against the proven benefit of PFO closure in preventing recurrent stroke in select patients.

## Case presentation

**Figure 1:**
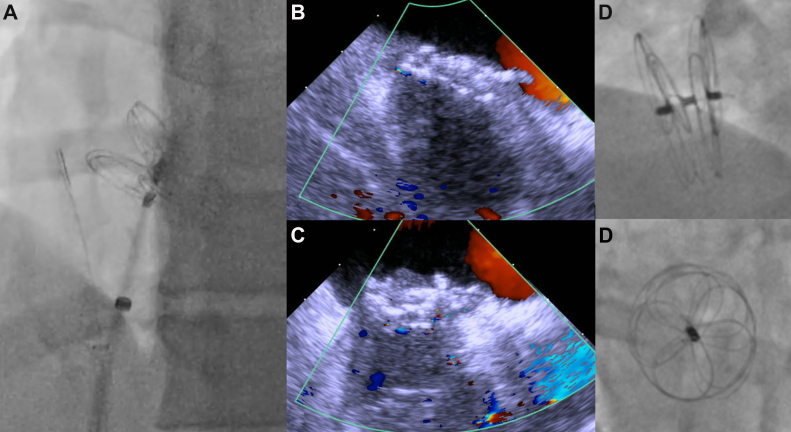
**Intraoperative imaging from PFO closure with the GORE CARDIOFORM Septal Occluder.** (A) ICE-guided deployment of the device. (B) Final color interrogation prior to device release. (C) No residual shunting after device release. (D) Final angiographic images after device release, showing adequate disk separation and a stable position.

**Figure 2:**
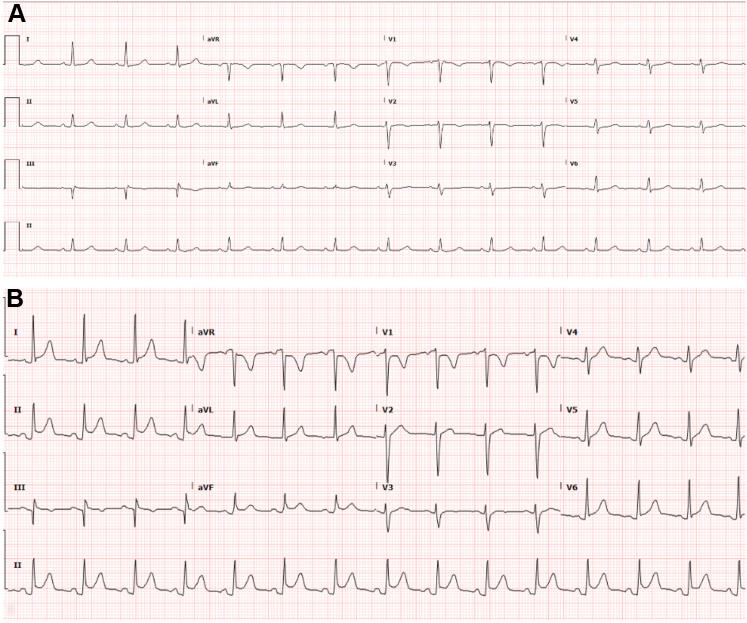
**Initial normal ECG and repeat ECG which shows findings indicative of pericarditis.** (A) Postprocedure ECG, which shows normal sinus rhythm. (B) Later ECG after representation to the hospital, which shows diffuse concave ST-segment elevations, PR segment depression, and reciprocal PR elevation in aVR.

**Figure 3:**
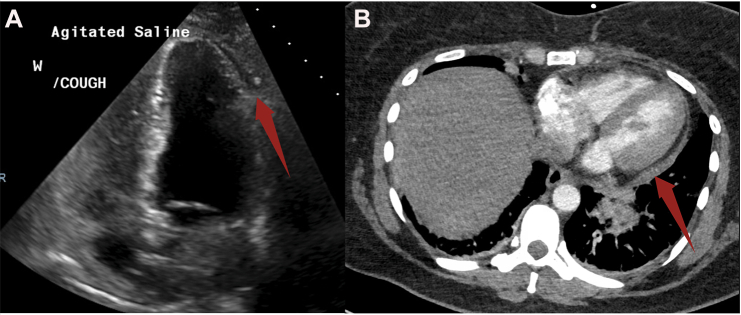
**Imaging demonstrating trace pericardial effusion.** Postprocedure imaging demonstrating a trace pericardial effusion by transthoracic echocardiogram (TTE) (A) and computed tomography angiography (B).

A 43-year-old woman with a history of cryptogenic stroke was found to have a large aneurysmal patent foramen ovale (PFO). She underwent elective, successful intracardiac echocardiography (ICE)-guided closure with a 30 mm GORE CARDIOFORM Septal Occluder (Figure 1A-D). Immediately postprocedure, she developed left-sided, substernal chest pain. Electrocardiogram (ECG) showed normal sinus rhythm (Figure 2A) and laboratory evaluation revealed only mild elevations in her white blood cell count (12.4 K/μL) and high-sensitivity troponin level (35.7 pg/mL). No intraprocedural complications were noted, and a transthoracic echocardiogram confirmed correct device positioning without wall motion abnormalities or pericardial effusion. She was discharged the following morning on aspirin and clopidogrel. Later that day, she returned to the emergency department with persistent sharp, pleuritic, and positional chest pain associated with dyspnea, unrelieved by acetaminophen or ibuprofen. Repeat workup revealed worsening leukocytosis (17.0 K/μL), marked troponin elevation (peaked at 8559.4 pg/mL), elevated inflammatory markers (C-reactive protein 119.9 mg/L, erythrocyte sedimentation rate of 24 mm/h), diffuse concave ST-segment elevations on ECG (Figure 2B), and a trace pericardial effusion on both computed tomography angiography (Figure 3A) and transthoracic echocardiogram (Figure 3B)—findings concerning for myopericarditis. COVID-19 testing was negative during her initial admission, and she exhibited no other symptoms of viral illness; hence, additional viral testing was deferred. On further questioning, the patient reported prior skin reactions to nickel jewelry, although no formal allergy testing was conducted pre- or postprocedure. She was treated with colchicine and ibuprofen, resulting in rapid symptom improvement. At the 3-month follow-up, cardiac magnetic resonance imaging showed no residual inflammation, and additional autoimmune serologies (antinuclear antibodies, rheumatoid factor, anti-SS-A/SS-B antibodies) were negative.

## Discussion

Myopericarditis refers to concurrent inflammation of the pericardium and myocardium, most commonly caused by viral infections but occasionally triggered by cardiac interventions. Although rare, pericarditis and myocarditis have been reported following intracardiac device implantation, including PFO closure.^1^^,^^2^ Proposed mechanisms include postcardiac injury syndrome (PCIS), an autoimmune reaction to pericardial trauma—which may occur during catheter-based interventions—and type IV hypersensitivity reactions to nickel, a component of nitinol used in all PFO closure devices.^1^^,^^3^^,^^4^ PCIS typically presents 1 to 6 weeks postprocedure and is usually limited to pericardial and pleural involvement.^4^ Our patient’s acute 36-hour onset and marked troponin elevation, indicative of significant myocardial involvement, make PCIS less likely. Moreover, the lack of intraprocedural complications (eg, from ICE manipulation), combined with the patient’s history of nickel sensitivity, further supports a delayed hypersensitivity reaction. The GORE CARDIOFORM Septal Occluder, although containing nitinol, is encapsulated in expanded polytetrafluoroethylene, which may reduce direct nickel exposure compared to devices like the AMPLATZER PFO Occluder (Abbott). However, no significant difference in the incidence of hypersensitivity reactions between devices has been established.^5^ Skin patch testing for nickel hypersensitivity has been proposed, and some studies have demonstrated an association with device-related complications, although the results have been mixed.^5^^,^^6^ Even in patients with known nickel sensitivity, the clinical relevance remains uncertain, as there are no nickel-free devices currently available on the market.^7^ The experimental Noblestitch EL system (KwiKnot) represents a nickel-free alternative to PFO closure. It uses two polypropylene sutures to approximate the septum primum and septum secundum, secured with a delivery and sealing system.^8^ Early results are promising, but data on long-term efficacy and safety are limited.^9^ Ultimately, the potential risk of hypersensitivity reactions must be weighed against the proven benefit of PFO closure in reducing stroke risk in select patients.
